# An updated review of SARS‐CoV‐2 detection methods in the context of a novel coronavirus pandemic

**DOI:** 10.1002/btm2.10356

**Published:** 2022-06-22

**Authors:** Yuxuan Zhang, Zhiwei Huang, Jiajie Zhu, Chaonan Li, Zhongbiao Fang, Keda Chen, Yanjun Zhang

**Affiliations:** ^1^ Shulan International Medical College, Zhejiang Shuren University Hangzhou China; ^2^ School of Laboratory Medicine and Life Sciences Wenzhou Medical University Wenzhou China; ^3^ Zhejiang Provincial Center for Disease Control and Prevention Hangzhou China

**Keywords:** nucleic acid molecular test, SARS‐CoV‐2, serological test, test kit evaluation, viral variants, virus detection

## Abstract

The World Health Organization has reported approximately 430 million confirmed cases of coronavirus disease 2019 (COVID‐19), caused by severe acute respiratory syndrome coronavirus 2 (SARS‐CoV‐2), worldwide, including nearly 6 million deaths, since its initial appearance in China in 2019. While the number of diagnosed cases continues to increase, the need for technologies that can accurately and rapidly detect SARS‐CoV‐2 virus infection at early phases continues to grow, and the Federal Drug Administration (FDA) has licensed emergency use authorizations (EUAs) for virtually hundreds of diagnostic tests based on nucleic acid molecules and antigen–antibody serology assays. Among them, the quantitative real‐time reverse transcription PCR (qRT‐PCR) assay is considered the gold standard for early phase virus detection. Unfortunately, qRT‐PCR still suffers from disadvantages such as the complex test process and the occurrence of false negatives; therefore, new nucleic acid detection devices and serological testing technologies are being developed. However, because of the emergence of strongly infectious mutants of the new coronavirus, such as Alpha (B.1.1.7), Delta (B.1.617.2), and Omicron (B.1.1.529), the need for the specific detection of mutant strains is also increasing. Therefore, this article reviews nucleic acid‐ and antigen–antibody‐based serological assays, and compares the performance of some of the most recent FDA‐approved and literature‐reported assays and associated kits for the specific testing of new coronavirus variants.

## 1 INTRODUCTION

On December 31, 2019, novel severe acute respiratory syndrome coronavirus‐2 (SARS‐CoV‐2), causing coronavirus disease 2019 (COVID‐19), was first identified in China, which has spread worldwide and caused a serious outbreak in a short period.^1^, ^2^ The World Health Organization (WHO) officially declared COVID‐19 as a public health emergency of international concern on January 30, 2020. WHO reports that there are currently nearly 430 million confirmed cases, approximately 6 million deaths, as well as nearly 10.4 billion vaccinations.

Coronaviruses belong to the coronaviridae family of the order Nidoviridae, comprising a set of enveloped viruses with a single‐stranded RNA genome (26–32 kb).^3^ There are four genera of coronaviruses, α, β, γ, and δ, and both SARS‐CoV‐2 and SARS‐CoV are members of the beta coronavirus family, while Middle East respiratory syndrome coronavirus (MERS‐CoV) belongs to family C of the genus β coronavirus. SARS‐CoV‐2 and SARS‐CoV share 79.6% sequence similarity, and research has revealed that these two viruses share the same vascular angiotensin‐converting enzyme 2 (ACE2) receptor for infection of human cells.^4^ SARS‐CoV‐2 is circulated primarily through breathing or contact with droplets from an infected person, with a latency period of about 2–14 days.^5^ The patient's clinical presentation after infection varies from asymptomatic to severe, with most infections not being severe.^6^ The leading causes of death commonly associated with COVID‐19 are respiratory failure, followed by septic shock, renal failure, hemorrhage, and cardiac failure.^7^

**FIGURE 1:**
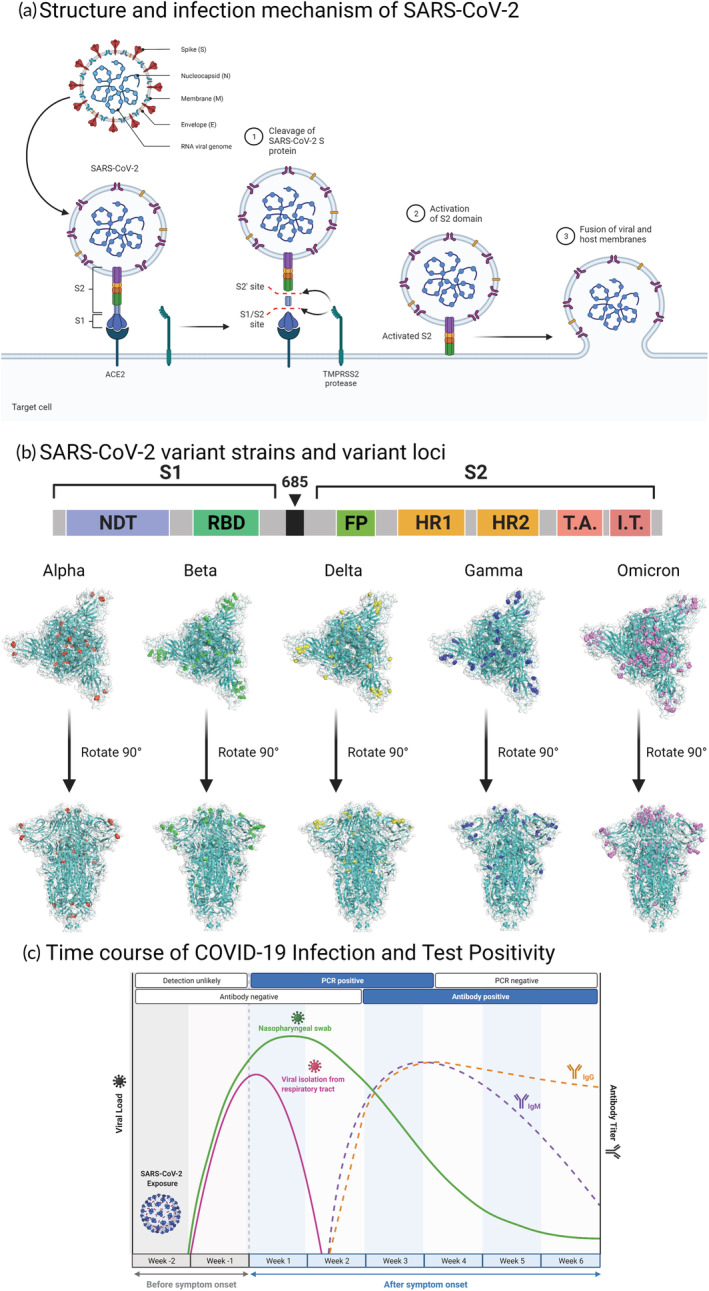
Biology and serology of SARS‐CoV‐2 infection (a) Structure and infection: SARS‐CoV‐2 is an RNA virus that consists of four structural proteins, the Spike (S) protein, Nucleocapsid (N) protein, Membrane (M) protein, and Envelope (e), together with many non‐structural proteins to maintain the biological traits of the virus. Step 1–3: S protein allows the virus to bind and enter human cells and consists of S1 and S2 subunits. S1 can bind the angiotensin‐converting enzyme 2 (ACE2) receptor. After S1 binds to ACE2, S protein is hydrolyzed by the action of TMPRSS2 protease. The activated S2 subunit can then further mediate the fusion of membranes between the host cell and the virus, allowing the virus to enter the host cell. (b) SARS‐CoV‐2 variants: S protein of the first Wuhan‐Hu‐1 strain consisted of 1273 amino acid residues, in which the S1 and S2 fragments are linked by amino acid bridges, S1 includes the N‐terminal domain (NTD) and receptor‐binding domain (RBD), and S2 includes the fusion peptide (FP), heptad repeat 1 (HR1), heptad repeat 2 (HR2), and other structures. Since the start of the outbreak, many strongly infectious SARS‐CoV‐2 mutant strains have emerged, such as B.1.1.7 (Alpha), B.1.351 (Beta), P.1 (Gamma), B.1.617.2 (Delta), and B.1.1.529 (Omicron), among which mutations are particularly common in the S protein and have a substantial effect on the infectivity of the virus. (PDB ID:7DDD) (c) Immunity: Following viral infection in humans, specific antibody reactions often appear between days 5 and 15 after infection, with the IgM response lasting 3–6 weeks and the IgG response lasting several months.

Thousands of cumulative mutations of the SARS‐CoV‐2 have occurred since its emergence, which often occurs naturally during replication. Many mutant strains such as Alpha (B.1.1.7), Beta (B.1.351), Gamma (P.1), Delta (B.1.617.2), and Omicron (B.1.1.529) have emerged, and the S protein, an important protein that facilitates virus transmission and entry into cells, has probably undergone more than 4000 mutations in its gene.^8^ Mutations in the receptor‐binding domain (RBD) region on the S proteins have also been shown recently to make the mutant strains more infectious,^9^ thus requiring techniques and devices that can detect mutant strains to control the development of outbreaks (Figure 1b).

With the sequencing of the virus genome and serological analysis of neutralizing antibodies (NAbs) among virus‐positive patients and recuperating patients, several kits based on nucleic acid molecular biology and antigen–antibody serology have been developed to assay the virus in swabs and blood specimens. Nucleic acid‐based assays include reverse transcription‐polymerase chain reaction (RT‐PCR), loop‐mediated isothermal amplification (LAMP), and clustered regularly interspaced short palindromic repeats (CRISPR)/CRISPR associated protein (Cas) systems; and serological immunoassays include enzyme‐linked immunosorbent assays (ELISAs), chemical immunoluminescence, and lateral flow immunoassays. However, RT‐PCR and ELISA, although considered the gold standards for molecular and serological assays of SARS‐CoV‐2, still have many problems, such as high cost and high time‐consumption, rendering them unable to implement rapid and highly sensitive testing in the face of a pandemic of SARS‐CoV‐2 and its variants, especially when the occurrence of viral mutations can affect primer or antibody binding.^10^ Therefore, rapid point‐of‐care (POC) detection techniques with high detection rates are being developed, such as the easier‐to‐operate loop‐mediated isothermal amplification (LAMP) and specific high‐sensitivity enzymatic reporter unlocking (SHERLOCK), based on recombinase polymerase amplification (RPA) and CRISPR/Cas, which requires only 0.5–1 h for highly specific detection and can detect mutant strains by designing primers that target their mutation sites. In addition, with the development of identifiable conserved protein tag tails, the detection rate of POC‐based immunoassay assays is also increasing. The development of POC assays is expected to be applied in the future in communities, rural areas, and other relatively poorly resourced areas for effective epidemic control.^11^ Moreover, the optimization of samples and swabs and other sampling tools, as well as the combination of artificial intelligence and deep learning networks, are also worth considering in the development of POC assays.

Herein, we comprehensively review the practical techniques designed to detect SARS‐CoV‐2, evaluate the results of relevant technologies (Table 1), and enumerate the relevant FDA‐approved test kits and the latest mutant detection devices.

## 2 STRUCTURE AND DETECTION OF SARS‐COV‐2

### 2.1 The structure and biology of SARS‐CoV‐2

Severe acute respiratory syndrome coronavirus 2 (SARS‐CoV‐2) is in the genus beta coronavirus and is the seventh coronavirus to infect humankind and cause acute respiratory disease.^12^ SARS‐CoV‐2 is 60–140 nm in diameter and comprises a single‐stranded positive‐sense RNA genome, capsid protein, and outer membrane assembly. Its genome size is from 29.8 to 29.9 kb and it includes 14 open reading frames (ORFs), which encode 27 proteins.^13^ Its genome is almost 80% homologous to SARS‐CoV and is similar to bat coronavirus (bat CoV), with 96% sequence similarity. Among the ORFs, ORF1ab, located in the 5′‐untranslated region (UTR), is the largest gene, encoding a variety of proteins required for viral transcription and replication, including multiple nonstructural proteins (NSP). The gene located in the 3′‐UTR encodes four predominant structural proteins, including the spike (S) protein, the membrane (M) protein, the envelope (E) protein, and the nucleocapsid (N) protein, and also encodes many nonstructural proteins.^14^ Among the four structural proteins, the S protein serves as a transmembrane protein that can mediate coronavirus entrance into the host cell by interacting with angiotensin‐converting enzyme 2 (ACE2).^15^ The M protein plays a role in determining the configuration of the viral envelope and the assembly of viral particles, and also counteracts the innate antiviral immune response triggered by viral RNA.^16^ The N proteins can combine with the RNA genome of viruses to constitute N protein–RNA complexes that participate in the replication cycle of the virus, the host response to viral infection, and genomic signaling. Meanwhile, the E protein, as the minimal major structural protein, can interact with host cell membrane proteins to participate in the viral production and the maturation process.^17^

### 2.2 Infection and sample collection of SARS‐CoV‐2

The S protein is a trimeric class I viral fusion protein that has a critical function in mediating the adhesion, fusion, and entry of SARS‐CoV‐2 into the human body.^18^, ^19^, ^20^ S protein has two subunits, S1 and S2. The S1 subunit can bind to the ACE2 receptor on the host cell and contains both an N‐terminal domain (NTD) and a receptor‐binding domain (RBD). The RBD of the S1 subunit carries out a hinge‐like motion when binding to the ACE2 receptor on the host cell membrane; the S2 subunit mediates the fusion of the host cell and the virus, and consists of the fusion peptide (FP), heptad repeat 1 (HR1), central helix (CH), connector domain (CD), heptad repeat 2 (HR2), transmembrane domain (TM), and cytoplasmic tail (CT).^15^, ^21^ The S1/S2 protease cleavage site exists between the S1 and S2 subunits, and the host protease can cleave the S protein at the S2′ site, which activates the protein and fuses the virus to the host cell membrane through irreversible conformational changes (Figure 1a).^22^

SARS‐CoV‐2 is extraordinarily stable at 4°C for 14 days and can be viable at 37°C for 24 h.^23^ It can be transmitted by respiratory secretions, aerosols, direct contact, the fecal‐oral route, mother‐to‐child transmission, and ocular transmission.^24^, ^25^ Infected individuals usually begin to show symptoms within 8.2–15.6 days, with an average of 11.2 days, with the disease progressing more rapidly in the elderly than in younger people.^26^ After human infection, the virus deposits in the upper respiratory tract and gradually penetrates deep into the lungs; however, the virus can also cause damage to the nervous system (e.g., the brain), digestive system (e.g., the liver, stomach, intestines), the urinary system (e.g., the kidneys), and the cardiovascular system.^27^

Viruses can provoke an immune reaction in the body, with immunoglobulin M (IgM) as the first line of protection, usually appearing within 3–5 days after infection. Immunoglobulin G (IgG) often appears 1 week after infection, with high affinity and adaptive response, and a long duration, making it useful as a marker of the previous infection (Figure 1c). There are two principal categories of SARS‐CoV‐2 tests adopted currently: (1) Nucleic acid‐based viral tests; and (2) antigen‐ and antibody‐based serological viral tests. The main specimens used are taken from the upper respiratory tract, lower respiratory tract, and blood. Sometimes, digestive tract samples are also used. Upper airway specimens mainly comprise nasopharyngeal swabs (NPS), oropharyngeal swabs (OPS), tongue swabs (LS), and mouthwash samples; lower respiratory tract samples mainly comprise sputum, tracheal inhalation (TA), and bronchoalveolar lavage fluid (BALF); blood samples can be whole blood or serum according to different test kits, and digestive tract samples often comprise anal swabs.^28^

### 2.3 Optimization of samples: virus collection and harvesting has an impact on detection results

The area sampled can have an impact on viral load, with samples collected from different sites having different viral loads. Upper respiratory tract samples are more common and nasopharyngeal swabs are considered to have the maximum viral load in diagnostic tests for respiratory viruses, including SARS‐CoV‐2.^29^ However, recently, researchers have analyzed saliva specimens and found that they are more sensitive than nasopharyngeal swabs (NPS) in the diagnosis of asymptomatic and mild coronavirus infections in children and adults.^28^, ^30^

The sampling method and choice of lysate also have a large impact on the detection, and the size of the swab end cotton balls and self‐sampling by health professionals versus the general population can also have an impact on the viral load. The WHO currently recommends that the gathered swabs are placed in the collection tubes containing virus transport media (VTM), Amies transport media, or sterile saline. Some scholars have used lysis buffer instead of virus storage solution to improve the security, sensitivity, and speed of the assay,^31^ while other researchers have developed a technique called Precipitation Enhanced Analyte Retrieval (PEARL) lysis solution that can rapidly isolate RNA, DNA, and proteins from a variety of sources in a sample and have high sensitivity, low cost, and simple operation for use in POC.^32^

When evaluating reagents from different companies, our group found that cross‐use of different brands of lysates affected the results of the assay, indicating that different brands of lysates need further optimization and validation.

## 3 NUCLEIC ACID‐BASED SARS‐COV‐2 DETECTION

### 3.1 Quantitative real‐time reverse transcription PCR: Detection principle and evaluation

#### 3.1.1 Detection principle, target, and process of qRT‐PCR

**FIGURE 2:**
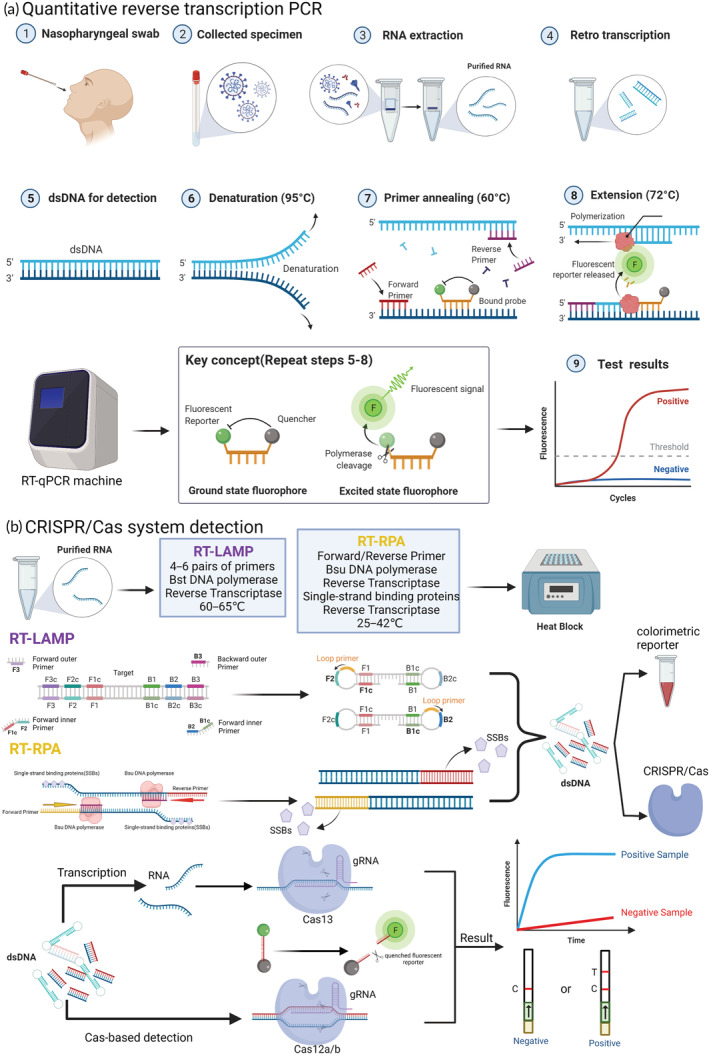
Nucleic acid‐based detection of SARS‐CoV‐2 (a) qRT‐PCR: Step 1–4: SARS‐CoV‐2 RNA in different collected samples, such as nasopharyngeal swabs, can be extracted and purified using an RNA extraction kit, and complementary DNA (cDNA) for amplification and detection can be obtained by reverse transcriptase; Step 5–9: template cDNA undergoes denaturation, primer annealing, and extension in the real‐time PCR instrument The fluorescence signal is released when the fluorescence molecule is no longer inhibited by the quenching molecule, and the instrument can convert the fluorescence signal in the cycle into the cycle threshold (CT) value, which can be expressed as the quantified viral load data, and the validity of SARS‐CoV‐2 infection is verified by comparison with negative controls and threshold lines. (b) CRISPR/Cas system: Based on reverse transcription recombinant polymerase amplification (RT‐RPA) and reverse transcription loop‐mediated isothermal amplification (RT‐LAMP), purified RNA can be amplified in an isothermal instrument, and the amplified product can be reported both by the chromogenic substances in the amplification system and by the CRISPR/Cas system for further specific cleavage of nucleic acids and determination of virus infection. The CRISPR‐associated Cas protein then binds to the guide RNA, forming a complex that can target cleavage of the viral nucleic acid sequence, and the result can be reported by the fluorescence quenching molecules in the reaction, by reporting the fluorescence signal, or by the side stream chromatography color development strip of the cleaved nucleic acid fragment.

Nucleic acid‐based assays are important tools to diagnosis viral infections, and polymerase chain reaction (PCR) is considered to be the “gold standard method” for virus detection because of its fast recognition, high sensitivity, and high specificity. The WHO and the FDA suggested the use of reverse transcription PCR (RT‐PCR), part of the Nucleic Acid Amplification Test (NAAT), which can be used to test for viruses.^33^ In the qRT‐PCR protocol, reverse transcriptase converts the extracted and purified SARS‐CoV‐2 RNA into cDNA, which is then amplified using gene‐specific primers in the quantitative real‐time PCR step of the qRT‐PCR protocol. Repeated thermal cycling in which the probe reports a fluorescent signal each time the target region of the genome is amplified results in quantitative detection (Figure 2a).^34^

Viral RNA extraction is now commonly performed by making use of upper airway specimens (e.g., nasopharyngeal swabs or oropharyngeal swabs, which were used more frequently) and lower airway specimens (e.g., phlegm and bronchoalveolar lavage fluid), but also blood, stool, and tissue samples. qRT‐PCR can target regions such as ORF1ab (RdRp), N, E, S, and ORF8 genes, among which the RdR1ab located in RdRP, and the N and E gene in OFR1ab are more conserved, with the detection of the RdRP and E genes being less restrictive and more sensitive compared with N gene detection.^35^ The WHO developed and shared primers that target the E gene, as well as the RdRp gene sequence, to screen for and confirm SARS‐CoV‐2 for the first time worldwide, and the design method based on this also successfully differentiated SARS‐CoV and SARS‐CoV‐2.^36^ CDC China also designed primers targeting the N gene and ORF1ab for inspection of viral RNA.

#### 3.1.2 Evaluation of qRT‐PCR detection results

The FDA has granted approval for well over 200 molecular diagnostic devices, and all approved qRT‐PCR devices can be used to report positive/negative results. Moreover, the amplification of viral RNA during the assay is graphically represented as a quantitative cycle, which is usually reported as a cycle threshold (CT) value.^37^ It has been reported that usually appropriate CT values range from 25 to 28, and when CT values exceed 28, nonspecific precipitating sequences are usually detected, and inactivation of Taq polymerase might also lead to different results. Clinical samples are usually identified as positive under two conditions: (1) The amplification signal/cycle of the sample needs to exceed the set threshold line during the positive cycle compared with that of the control; (2) having a relatively lower CT value/number, where the CT value is inversely proportionate to the quantity of RNA/DNA in the given specimen. During the detection process, CT values are influenced by sample type, RNA extraction, and the qRT‐PCR kits and equipment. The CT values of various clinical samples during actual diagnosis vary between 16.9 and 38.8, and Ct values <40 are often suggested as indicators of SARS‐CoV‐2 positivity.^38^

However, false‐negative results of qRT‐PCR often interfere with the control of virus transmission epidemics, and different samples and insufficient viral load of collected samples are an important factor contributing to false negatives. The overall sensitivity of nasopharyngeal swabs and nasopharyngeal aspirate samples in RT‐PCR was reported to be in the range of 45%–60%.^39^ A study of 213 patients with new coronary pneumonia within the first 7 days showed false‐negative rates of 11%, 27%, and 40% for sputum, nasal swabs, and oral swabs, respectively. The timing of sampling before and after symptom onset is also an important factor in the generation of false negatives, and the false‐negative rate varies over time; Kucieka et al. used a Bayesian hierarchical model to analyze 1330 confirmed cases to assess the false‐negative rate between 5 days before symptom onset and 21 days after the occurrence of symptoms, and found that the false‐negative rate on the day before the symptoms appear, the day symptoms appear, and the 21st day after symptom onset were 67%, 38%, and 66%, and the median false‐negative rate gradually decreased to 20% on days 3 and 4 of symptom onset.^40^ In addition, the presence of false positives can interfere with the determination of the true disease status of patients; therefore, some investigators have suggested the use of multiplex combinations or RT‐PCR combined with serology during infection to control the false‐negative and false‐positive rates.

There remain some gaps between different RT‐PCR kits in terms of specificity and sensitivity, depending on their targets, primer design, and other factors, and many institutions and laboratories have analyzed and assessed the effectiveness of different RT‐PCR kits. Recently, Chinese researchers assessed the effectiveness of five RT‐PCR kits from Da An, Liferiver, Kinghawk, among which Da An (detecting ORF1ab, N) had 100% good specificity with a limit of detection (LoD) of 250 copies/ml. Ninety‐six samples were used by Altamimi et al. at the Saudi Center for Disease Prevention and Control (SCDC) for the analysis of TIB MOLBIOL, Altona Diagnostics, Thermo Fisher Scientific, and other 12 different commercially available RT‐PCR kits for SARS‐CoV‐2. The results showed that except for the LYRA kit, which had a sensitivity of only about 66.6%, all kits had a sensitivity between 95% and 100%, with the BGI, IQ Real, Sansure, and RADI kits being the most sensitive (100%). The specificity of most of the kits was 100%, except for four kits, BGI, KAIRA, PowerCheck, and Sansure, which were around 97%. Altamimi et al. also found that the design of the primers had a large impact on the performance of the kits.^41^ Kim et al. tested the Allplex SARS‐CoV‐2/FluA/FluB/RSV assay (Seegene), Standard M nCoV real ‐time detection kit (SD Bi) for SARS‐CoV‐2 and its variant B.1.351 (Beta)‐time detection kit (SD Biosensor), and U‐TOP COVID‐19 detection kit (Seasun Biomaterials). The LoD for the target genes was estimated to be 1300 copies/ml for the latter three kits and 650 copies/ml for the Allplex SARS‐CoV‐2/FluA/RSV kit. The Standard M nCoV real‐time detection kit had 100% specificity and sensitivity, and was the best one for RdRp gene detection.^42^ In a study with 354 patients with COVID‐19 pneumonia as a random sample source, the clinical performance of three test kits, Sansure Biotech, GeneFinderTM, and TaqPathTM, were evaluated, revealing LOD values of 200 copies/ml, 500 copies/ml, and 10 genomic copy equivalents, respectively, with Sansure Biotech having the highest specificity and sensitivity.^43^ RT‐PCR analysis for mutant loci is quite important to control outbreaks of mutant strains, and a total of five RT‐PCR assays for relevant mutation loci, such as SARS‐CoV‐2 Variants II Assay Allplex, UltraGene Assay SARS‐CoV‐2452R & 484K & 484Q Mutations V1 RT‐PCR assay kits were evaluated. The overall mean Ct value of the five kits was 23.6 ± 3.8, with accuracy ranging from 96.9% to 100%, among which the SARS‐CoV‐2 Variants II Assay Allplex (for L452R, W152C, K417T, K417N) kits had 100% sensitivity and specificity.^44^

#### 3.1.3 PCR technique used in variants detection

Outbreaks are difficult to control because of the high infectivity of mutant strains, and the emergence of mutant strains can adversely affect the performance of molecular assays, especially those targeting genomic single‐target tests. N and E genes as targets, while the S gene is often off‐target due to its susceptibility to mutation. In one study, a mutation in the viral genome at locus 26,340, C to U, caused a failure of the cobas SARS‐CoV‐2 E gene qRT‐PCR assay, but because the detection probe of the cobas SARS‐CoV‐2 qRT‐PCR kit can target both regions of the genome, the experimenter was still tested positive, which also reminded researchers to develop Multiple‐target primer sets were developed to avoid false‐negative results.^45^ Specific tracking of mutant strains can also be achieved by sequencing emerging mutant strains and changing the corresponding primer and probe sets, and many devices have been developed specifically to detect mutant strains, particularly those based on detecting genetic loci where the S protein is more susceptible to mutation.

RT‐PCR analysis for mutant loci is quite important to control outbreaks of mutant strains, and a total of five RT‐PCR assays for relevant mutation loci, such as SARS‐CoV‐2 Variants II Assay Allplex, UltraGene Assay SARS‐CoV‐2452R & 484K & 484Q Mutations V1 RT‐PCR assay kits were evaluated. The overall mean Ct value of the five kits was 23.6 ± 3.8, with accuracy ranging from 96.9% to 100%, among which the SARS‐CoV‐2 Variants II Assay Allplex (for L452R, W152C, K417T, and K417N) kits had 100% sensitivity and specificity.^44^ Novel whole‐genome sequencing technologies based on the EasySeqTM RC‐PCR SARS‐CoV‐2 WGS kit and RT‐PCR have proven to be useful for high‐throughput detection of mutant strains of SARS‐CoV‐2.^46^ Vega‐Magaña et al. designed three specific primers and probes for qRT‐PCR detection based on the N501Y, 69‐70del, K417N, and E484K S mutations, which played an important role in detecting the E484K mutation and P.2 mutant strains.^47^ Exploiting the good selectivity and self‐quenching properties ascribed to molecular beacons, researchers developed a two‐tube multiplex qRT‐PCR detection method that can identify present viruses of concern (VOCs) via the detection of eight different mutation sites in the S protein.^48^ Based on Multiplex PCR‐Mass Spectrometry (MS) Minisequencing Technology, Zhao et al. established a matrix‐assisted laser desorption/ionization (MALDI)‐time of flight (TOF) MS technique based on multiplex PCR amplification products using nucleic acid sequences of SARS‐CoV‐2 nonmutants and synthetic plasmids carrying mutants, which can detect, for example, HV6970del, N501Y, and K417N, in seven mutation loci of the S protein RBD region and nine other combined variant types, effectively detecting B.1.1.7 (Alpha), B.1.351 (Beta), B.1.429 (Epsilon), B.1.526 (Iota), P.1 (Gamma), and B.1.617.2 (Delta).^49^

### 3.2 Reverse transcription loop‐mediated isothermal amplification

#### 3.2.1 Detection principle, target, and process of RT‐LAMP

LAMP, as a new DNA/RNA amplification technique, does not require expensive thermal cyclers (unlike PCR). LAMP allows isothermal amplification in resource‐limited areas with the advantages of high speed, sensitivity, and specificity. RT‐LAMP, as a NAAT, can reverse transcribe the RNA in the sample to obtain cDNA, followed by automatic circular strand replacement DNA synthesis by 4–6 internal and external primers to form a dumbbell DNA structure with the participation of Bst DNA polymerase.^33^, ^50^ The nucleic acid amplification stage requires four to six primers to amplify the nucleic acid at a stable temperature of 60–65°C in combination with six regions of the target gene,^51^, ^52^ where four primers are necessary for the LAMP reaction (internal, external, forward, and reverse); however, more primers can improve the sensitivity and specificity of the assay, and significantly decrease the time required for the assay.

**TABLE 1:** Evaluation of the advantages and disadvantages of SARS‐CoV‐2 detection technology

Subjects that based	Method	Reaction time	Advantages	Disadvantages
Diagnostic Medical Imaging	CT	About 1 h	More accurate in determining disease status	Cannot be distinguished from other viral pneumonia
	Artificial intelligence: CT combined with algorithm‐based deep learning	Same as CT	Diagnostic capability based on continuous optimization of algorithms	AI recognition models need to pass a certain time in training, and the technical requirements are high
Nucleic acid‐based molecular biology diagnostics	Next‐generation sequencing (NGS)	1–2 days	Can display the complete genome and effectively identify mutant strains	Need for well‐equipped laboratories and knowledgeable laboratory staff
	qRT‐PCR	1–2 days	Gold standard: High specificity and sensitivity Quantitative and qualitative	High rate of false negatives, and has experimental operation and cost requirements
	RT‐LAMP	30–60 min	Simple reaction conditions, Suitable for point‐of‐care testing (POCT)	Primer design is complicated
	CRISPR‐Cas system	30–60 min	Suitable for point‐of‐care testing (POCT)	Possible “off‐target” phenomenon can affect the judgment of the test results
Serological diagnosis based on antigen–antibody	Colloidal gold immunolateral flow chromatography	15–20 min	Suitable for point‐of‐care testing (POCT), Result visualization	Window period exists for early detection, Cross‐reactivity with other viruses
	ELISA	4–6 h	Enables amplification of virus and antibody signals	Poor repeatability, Easy to contaminate

Common targets used for RT‐LAMP assays are similar to those of RT‐PCR and the ORF1ab, S, E, and N genes can be targeted for SARS‐CoV‐2.^53^, ^54^ Yan et al. developed an RT‐LAMP assay to analyze ORF1a and S genes in just 30 min, and all 130 clinical samples in the experiment showed 100% detection sensitivity and specificity.^54^ Primer‐probe targets against SARS‐CoV‐2 ORF1ab and S genes have also been reported.^54^ The ORF1b region was also selected for LAMP amplification using six primers and the results obtained were verified by gel electrophoresis.

#### 3.2.2 Evaluation of RT‐LAMP detection results

LAMP‐based assays are available in tiny PCR tubes, where dumbbell‐like structures with many DNA synthesis initiation sites can be transferred into longer tandems (where each tandem has many DNA synthesis initiation sites) during nucleic acid amplification, eventually leading to the accumulation of different DNA structures with the same target DNA sequence,^33^ which in turn can be determined by turbidity, the addition of pH‐sensitive dyes, or intercalation dyes to produce color or fluorescence; agarose gel electrophoresis of the products can also be used to determine SARS‐CoV‐2 infection.^55^ RT‐LAMP uses more primers than RT‐PCR; therefore, it has a higher specificity.^51^ The LAMP procedure is up to 10 times more sensitive than routine PCR for the assay of new coronaviruses in the absence of false negatives. Yu et al. also designed a LAMP‐based diagnosis technique for SARS‐CoV‐2 testing using six primers, termed iLACO (isothermal LAMP‐based method for COVID‐19), and found that the sensitivity and accuracy of iLACO were better than that of the Taqman‐based qPCR detection method.^56^ Recently, it was also shown that RT‐LAMP targeting the SARS‐CoV‐2 N gene could specifically detect viral RNA of SARS‐CoV‐2 without cross‐reactivity with related coronaviruses. (e.g., MERS‐CoV, HCoV‐229E) and other viruses that can lead to respiratory illnesses (e.g., RSVA, RSVB, and ADV).^57^ These results also suggest that RT‐LAMP‐based technology has a promising prospect in the diagnosis of SARS‐CoV‐2 infection.

The sensitivity and specificity of RT‐LAMP are usually compared with those of RT‐PCR, and Promlek et al. have performed screening and testing between RT‐LAMP and RT‐PCR kits and between different RT‐LAMP kits. In a recent comparative study with a sample of 315 nasopharyngeal swabs, investigators tested the FastProof 30 min‐TTR SARS‐CoV‐2 RT‐LAMP method against Sansure Novel Coronavirus (2019‐nCoV) Nucleic Acid Diagnostic Kit. The general sensitivity was 81.82% and the specificity of the RT‐LAMP kit was 100%, in which the RT‐LAMP sensitivity was 100% for samples with Ct values <31, but when Ct value was >36, this value decreased to as low as 15.79%, suggesting that a low viral load is associated with the poor sensitivity of RT‐LAMP.^58^ Jang et al. designed five sets of LAMP primers for the N, E, and RdRp genes, and evaluated and optimized the LoD of different primer combinations for LAMP using clinical nasopharyngeal swabs. Finally, the SARS CoV‐2 RdRP (FAM)/N (Cy5)/internal control RT‐LAMP assay indicated the lowest LOD and the sensitivities of this LAMP kit in comparison with the RT‐PCR kit (RdRP: 93.85%, N: 94.62% and RdRP/N: 96.92%) were slightly lower than that of the AllplexTM 2019‐nCoV assay (100% sensitivity for RdRP, E and N gene, and 97.69% sensitivity for IC), but better than the AllplexTM 2019‐nCoV assay (100% sensitivity for RdRP, E and N, and 97.69% sensitivity for IC). 97.69%), and the PowerChekTM 2019‐nCoV real‐time PCR kit (RdRP: 92.31%, E: 93.85% and RdRP/E: 95.38%).^59^ Dong et al. evaluated 19 RT‐LAMP assay kits using 4 standard RNAs and 29 clinical specimens. Six sets of primers showed the best results (Set‐4, 10, 11, 13, 14, and 17), which also showed high concordance (87.8%–97.6%), with Set‐4 having the maximum positive detection rate (82.8%) and a LOD of 3 copies per 25 μl reaction; thus, Set‐4 was recommended as the preferred diagnosis set for patients; researchers also recommended utilizing Set‐4 and any of Set‐10, 11, 13, and 14 for efficient POC‐based detection.^60^

### 3.3 SHERLOCK: A CRISPR‐Cas‐based SARS‐CoV‐2 detection method

Clustered regularly interspaced short palindromic repeat (CRISPR) technology is considered a robust instrument to modify genomes and can be used to easily alter nucleic acid sequences and gene functions. CRISPR in combination with CRISPR‐associated proteins (Cas) proteins has great potential to correct genetic defects, treat and prevent disease transmission, and in clinical research. The CRISPR‐Cas system makes a significant contribution to therapy as well as diagnosis for different infectious disease molecules, for example, CRISPR‐Cas9 could be used as an antiviral agent to treat HIV infection, in diagnostic tests for Zika virus, and for methicillin‐resistant *Staphylococcus* aureus infection.^61^ In recent years, research on guide RNA and RNA‐targeted CRISPR effectors has also laid the groundwork for diagnostics and suppression of RNA viruses based on CRISPR‐Cas13.^62^ CRISPR‐Cas can usually be classified into two categories, each of which contains specific types^63^: (1) A class comprising a complex structure consisting of RNA‐guided multi‐unit protein complexes that contain type I, type III, and type IV. (2) Type II is a single‐protein CRISPR system containing type II (recognized by the Cas9 enzyme), type V (recognized by Cas12a, C2c1, or C2c3 nucleases), and type VI (recognized by Cas13 effector enzymes), among which Cas12 and Cas13 are usually used for detection and therapy of viral diseases.^64^

Specific high‐sensitivity enzymatic reporter unlocking (SHERLOCK) is the first CRISPR/Cas13‐based technology, consisting of recombinase polymerase amplification (RPA) or RT‐RPA, as well as Cas13a.^65^ The complex formed recognizes and cleaves the target nucleic acid sequence, while nontarget RNAs in the reaction system that are coupled to fluorescent reporter molecules will snap off, the quenched molecule is released, and the fluorescent signal is visible, resulting in a rapid method to detect the targeted viruses, even at very low concentrations.^66^ SHERLOCK has been used to detect Zika and dengue viruses. These findings show good promise for SHERLOCK as a platform for the rapid, portable, and multiplex quantitative detection of emerging viral infections.^65^, ^67^ Zhang et al. combined RT‐LAMP with a CRISPR‐mediated assay to develop the STOPCovid assay, which does not require sample extraction, but instead lyses viral particles at room temperature (22°C) or in one pot using QuickExtract particles for 10 min. The authors also used a magnetic bead purification method to simplify RNA extraction and improve sensitivity, with the process using Cas12b belonging to the bacterium *Aphthous aliphaticus* (AapCas12b), which can maintain sufficient activity with LAMP (55–65°C) in the same temperature range for the N gene assay (Figure 2b).^68^

CRISPR/Cas technology also serves an integral role in the specific detection of mutant strains, and Liang et al. developed the CRISPR‐Cas12a technology based on the K417N/T, L452R/Q, T478K, E484K/Q, N501Y, and D614G mutant S loci. In comparison with RT‐PCR, the CRISPR‐Cas12a assay could distinguish four wild‐type viruses as well as the Alpha, Beta, and Delta variants of SARS‐CoV‐2.^69^ Liang et al. have also designed CRISPR RNAs specific for Omicron (crRNA‐S‐37X vs. crRNA‐S‐49X) and constructed CRISPR/Cas12a‐based detection kits for S371L, S373P, and S375F (corresponding to crRNA‐S‐37X), Q493R, G496S, and Q498R (corresponding to crRNA‐S‐49X) mutant loci were analyzed for the specific detection of Omicron.^70^ The POC‐based miSHERLOCK CRISPR/Cas suite for the S protein mutation sites N501Y, Y144del, and E484K was also demonstrated to detect Alpha, Beta, and Gamma variants.^71^

### 3.4 Analysis of nucleic acid‐based SARS‐CoV‐2 detection and other methods

Although real‐time RT‐qPCR is considered the gold standard method and the most widely applied in most countries, the detection protocols of all mentioned above need expensive experimental instruments, reagents, professional laboratories, and researchers. What's more, the accuracy of test results depends a lot on sample types^72^ and different detection targets.^7^ Therefore, this method is not suitable for POCT or somewhere deficient in medical resources. In contrast, RT‐LAMP does not need skilled researchers and specialized labs. The method costs only 30–60 min with high accuracy, which decreases the burden of sample transit and the risk of delayed reporting. Despite that many researchers develop different techniques based on RT‐LAMP targeted 4–6 primers, the impact of cross‐reaction and new coronavirus mutations hinder the development of its commercialization to some extent. Cas12 and Cas13, RNA‐guided components of the bacterial adaptive immune system, can target single‐ and double‐stranded DNA or single‐stranded (ss) RNA substrates, respectively.^73^, ^74^ Therefore, the CRISPR‐Cas system can be developed as a novel strategy to detect SARS‐CoV‐2 RNA rapidly. SHERLOCK was demonstrated that it can detect RNA and DNA of target diseases rapidly and accurately. Apart from SHERLOCK, Cas13 protein also can be used to detect SARS‐CoV‐2. The difference is that Cas13 exhibits cleavage is activated by ssRNA sequence bearing complementarity to its crRNA spacer instead of DNA target. So an additional T7 transcription is needed after amplification to convert the DNA amplicons to RNA.^75^ However, limited by PAM and PFS, the target sequence is only a short specific region, which is an obstacle for some short targets. Besides, developing multi‐channels test assays is a major trend in the future. But non‐specific collateral cleavage of Cas12 and Cas13 systems may influence other target pathogens, which is not conducive to developing multi‐channels tests.

In efforts to develop rapid diagnostic tests, more NAATs are investigated except RT‐LAMP and CRISPR‐Cas systems, such as transcription‐mediated amplification (TMA),^76^ nicking enzyme‐assisted reaction (NEAR),^77^ and recombinase polymerase amplification (RPA).^78^ NEAR can achieve a linear amplification of DNA template by two enzymes (nicking endonuclease and DNA polymerase), which reaction temperature will occur at 60°C. Compared with LAMP, the speed of amplification of RPA is increasingly faster at 37°C or less.^79^ Although these technologies are all potentially applied to POC applications, only TMA has been commercialized in a high‐throughput instrument.^77^ In the circumstance of pandemic COVID‐19, TMA can meet the need for pandemic‐scale diagnostic testing. Not only can the TMA system possess high efficiency, but also high sensitivity and specificity. Pham et al. demonstrated that the TMA assay achieved 95% positivity at 0.003 TCID50/ml in three specimen matrices(pooled NP swab specimens, STM, and saline) and was not caused any cross‐reaction in 30 nontarget viral, bacterial, and fungal microorganisms or 30 NP swab specimens.^76^ In summary, these NAATs show great potential for their simplicity, sensitivity, specificity, and low cost of time. It is hopeful that apply these NAATs into practice and develop multiplex detection of SARS‐CoV‐2, such as influenza A and B (Table 2).

**TABLE 2:** Summary of molecular diagnostic tests for SARS‐CoV‐2 (EUAs)

Detection target	Collected samples	Limit of detection (LoD)	Manufacturer	Detection principle	Diagnostic	Source
ORF1ab, E gene	individual or pooled nasal, nasopharyngeal, and oropharyngeal swab samples	Target 1 (SARS‐CoV‐2): 25 copies/ml (95% CI: 17–58 copies/ml) Target 2 (pan‐Sarbecovirus): 32 copies/ml (95% CI: 21–73 copies/ml)	Roche Molecular Systems, Inc. (RMS)	Real‐time RT‐PCR	Cobas SARS‐CoV‐2	^126^
ORF1ab and N gene	upper respiratory tract specimens	1 copy/μl	SEASUN BIOMATERIALS, Inc.	RT‐LAMP	AQ‐TOP COVID‐19 Rapid Detection Kit PLUS	^127^
	upper respiratory and BAL specimens	150 copies/ml	Euroimmun US, Inc.	Real‐time RT‐PCR	EURORealTime SARS‐Cov‐2	^128^
N gene, RNase P gene	nasopharyngeal, anterior nasal, mid‐turbinate, oropharyngeal swab specimens, nasopharyngeal wash/aspirate, and nasal aspirate specimens	640 GC/ml (Determination of SARS‐CoV‐2 heat‐inactivated virus) 40 GE/ml (Detection of quantified genomic viral RNA from SARS‐CoV‐2)	Becton, Dickinson and Company (BD)	Real‐time RT‐PCR	BD SARS‐CoV‐2 Reagents for BD MAX System	^129^
	nasopharyngeal swab, oropharyngeal swab, mid‐turbinate nasal swab, and anterior nasal swab specimens	0.125 copies/μl	Fluidigm Corporation	Real‐time RT‐PCR	Advanta Dx COVID‐19 EASE assay	^130^
	upper respiratory specimens	20 copies/μl	Mammoth Biosciences, Inc.	RT‐LAMP, CRISPR/Cas12	SARS‐CoV‐2 DETECTR Reagent Kit	^131^
	anterior nares specimens	250 copies/swab	LGC, Biosearch Technologies	End‐Point RT‐PCR	Biosearch Technologies SARS‐CoV‐2 ultra‐high‐throughput End‐Point RT‐PCR Test	^132^
	upper respiratory specimens	1.2 copies/μl	Exact Sciences Laboratories	Real‐time RT‐PCR	SARS‐CoV‐2 (N gene detection) Test	^133^
	anterior nasal swab specimens	6.25 copies/μl	Premier Medical Laboratory Services	Real‐time RT‐PCR	PMLS SARS‐CoV‐2 Assay	^134^
	nasopharyngeal and oropharyngeal swab specimens	1 copy/μl	Avellino Lab USA, Inc.	Real‐time RT‐PCR	AvellinoCoV2 test	^135^
OFR1ab, N gene, RNase P gene	nasopharyngeal swabs, and anterior or mid‐turbinate nasal swabs	Nasopharyngeal swab: 75 GCE/ml of sample Anterior nasal swab: 75 GCE/ml of sample	Thermo Fisher Scientific Inc.	Real‐time RT‐PCR	TaqPath COVID‐19 RNase P Combo Kit 2.0	^136^
	upper respiratory tract specimens and bronchoalveolar lavage specimens	ORF1ab target: 6.75 cp/μl VTM N target: 1.35 cp/μl VTM	Sherlock BioSciences, Inc.	Sherlock,RT‐LAMP, CRISPR	Sherlock CRISPR SARS‐CoV‐2 Kit	^137^
N and E gene	nasopharyngeal (NP), oropharyngeal (OP), mid‐turbinate (MT), and anterior nares (nasal) swabs	75 copies/μl	MobileDetect Bio Inc.	RT‐LAMP	MobileDetect Bio BCC19 (MD‐Bio BCC19) Test Kit	^138^
	upper respiratory specimens	0.0200 PFU/ml	Cepheid	Real‐time RT‐PCR	Xpert Xpress SARS‐CoV‐2 test	^139^
	saliva specimens	6400 GE/ml	MicroGEM U.S., Inc.	Real‐time RT‐PCR	MicroGEM Sal6830 SARS‐CoV‐2 Saliva Test	^140^
N gene	upper respiratory tract specimens	20 copies/μl	UCSF Health Clinical Laboratories, UCSF Clinical Labs at China Basin	RT‐LAMP, CRISPR/Cas12	SARS‐CoV‐2 RNA DETECTR Assay	^141^
	nasopharyngeal, anterior nasal, mid‐turbinate nasal, or oropharyngeal swab specimens	100 copies/ml (nasopharyngeal matrix)	Mammoth Biosciences, Inc	RT‐LAMP, CRISPR	DETECTR BOOST SARS‐CoV‐2 Reagent Kit	^142^
ORF1ab	anterior nasal swab samples	800 copies/ml	Detect, Inc.	LAMP, Lateral Flow Strips	Detect Covid‐19 Test	^143^
RdRP, E, and N genes	nasopharyngeal (NP), oropharyngeal (OP), bronchoalveolar lavage (BAL) specimens	140 copies/ml	PlexBio Co., Ltd.	RT‐PCR	IntelliPlex SARS‐CoV‐2 Detection Kit	^144^
N, ORF1ab, S gene, RNase P RNA (and DNA)	anterior nasal swab specimens	1 copy/μl	UCSD BCG EXCITE Lab	Real‐time RT‐PCR	UCSD EXCITE COVID‐19 EL Test	^145^

## 4 ANTIGEN–ANTIBODY‐BASED SEROLOGICAL SARS‐COV‐2 DETECTION

**FIGURE 3:**
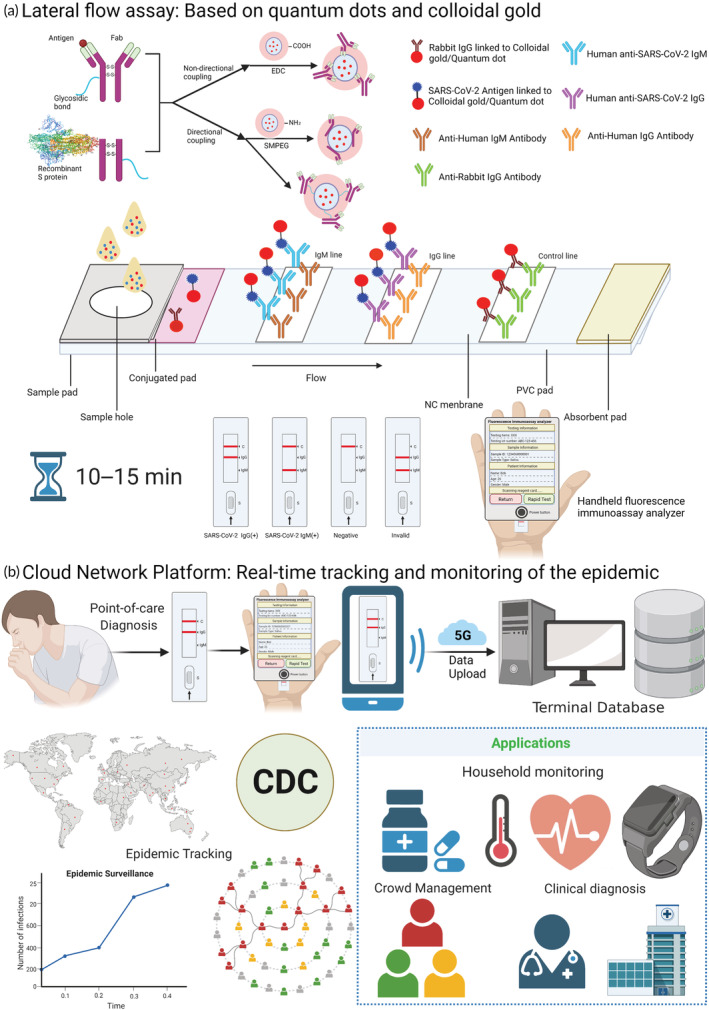
Serological detection of SARS‐CoV‐2 (a) Lateral flow assay: Quantum dots/colloidal gold can couple antibodies via specific labeling (using agent Maleamide–polyethylene glycol–succinimide ester (SMPEG)) and nonspecific labeling (using EDC/NHS chemistry methods). The rapid quantum dot and colloidal gold immunodiagnostic method for SARS‐CoV‐2 antibody‐based on high specificity recombinant protein and quantum dot/colloidal gold immunofluorescence probes by double antibody sandwich or indirect method methodology using lateral flow assay. The patient sample added to the sample pad will move to the absorbent pad along the NC membrane by chromatography, which will form the tagged‐antibody–antigen–antibody complex. After 10–15 min, test results can be observed on the test kit and operators can get an accurate fluorescence signal by a handheld fluorescent immunoanalyzer. (b) Cloud Network Platform: Rapid test kits can be used at the point of care for suspicious population screening tests, mobile devices such as cell phones can be used for result identification, handheld fluorescent immunoassay analyzers can perform a quantitative and qualitative analysis of test results, and qualitative and quantitative data can be uploaded to the terminal database, the CDC can manage relevant infections and suspicious populations through analysis of qualitative and quantitative data, give relevant clinical diagnosis recommendations, and combine with wearable devices such as smartwatches to achieve daily monitoring of people's medication, body temperature, heart rate, and other vital signs at the point of care such as communities and families, to control the development of epidemics in a timely and effective manner.

Although viral nucleotide‐based RT‐PCR assays have been the standard diagnostic approach to SARS‐CoV‐2 detection, RT‐PCR‐based test kits still have many problems: (1) PCR tests need to be accredited professional laboratories with high‐cost instruments and well‐trained laboratory personnel; (2) the test has a long turnaround period and is complex to perform, usually taking 2–3 h to obtain results; and (3) inappropriate false‐positive and false‐negative results resulting from external factors, such as the collected samples and handling, can occur (Figure 3).

The human body produces specific antibodies against SARS‐CoV‐2 infection, and these antibodies can be used as targets for the fast, simple, and highly sensitive detection of the virus with a sensitivity of >57.2% and up to 87.5% for IgM and >71.4% and up to 87.5% for IgG.^80^ Notably, the RBD of the S protein displays higher antigenicity than the N protein, as shown by studies showing sensitivities of 96.8%, 96.8%, and 98.6% for RBD IgM, IgG, and IgA, respectively.^81^ Many experts recommend the detection of specific antibodies as a supplement to nucleic acid testing, and paper‐based lateral flow immunoassays (LFIA) have been developed (Figure 3a).

**TABLE 3:** Summary of antigen–antibody‐based serology tests for SARS‐CoV‐2(EUAs)

Detection target	Collected samples	Clinical Performance	Manufacturer	Detection principle	Diagnostic	Source
IgM and IgG	Plasma, serum	PPA (≤6 days, 7–14 days, >14 Days, Days from Symptoms Onset to Blood Collection): IgM: 100%, 85.71%, 99.25%; IgG: 0.00%, 76.19%, 98.50%; NPA: IgM/IgG: 99.43%	Hangzhou Laihe Biotech Co., Ltd.	Lateral Flow (Colloidal Gold)	LYHER Novel Coronavirus (2019‐nCoV) IgM/IgG Antibody Combo Test Kit	^146^
	Human venous/fingerstick whole blood, serum, plasma	PPA (0–7 days, 8–14 days, ≥15 days): (1) Serum: IgM: 100%, 86.7%, 84%; IgG: 87.5%, 86.7%, 100% (2) Whole Blood: IgM: 100%, 100%, 100%; IgG: 100%,100%,100%; NPA: 99.04%	Assure Tech. (Hangzhou Co., Ltd)	Lateral Flow (Colloidal Gold)	Assure COVID‐19 IgG/IgM Rapid Test Device	^147^
	Human venous whole blood, plasma or serum	Either IgG+ or IgM+: PPA: 96.7% (95% CI: 90.7–98.9%) NPA: 97.0% (95% CI: 91.6–99.0%)	Healgen Scientific LLC	Lateral Flow (Colloidal Gold)	COVID‐19 IgG/IgM Rapid Test Cassette (Whole Blood/Serum/Plasma)	^148^
	Human serum and serum	Total IgM and IgG Combined PPA: 93.94% (≤ 7: 43.75%, 8–14: 93.40%, ≥15: 100%, Days post symptom onset) NPA: 98.67%	Shenzhen New Industries Biomedical Engineering Co., Ltd.	CLIA	MAGLUMI 2019‐nCoV IgM/IgG	^149^
	Human serum, acid citrate dextrose (ACD) plasma, fingerstick whole blood	PPA(0–7, 8–14, ≥ 15, Days of Symptoms Onset): IgM:76.9%, IgG:75.9% NPA: IgM:99.6%, IgG:99.3%	Megna Health, Inc.	Lateral Flow	Rapid COVID‐19 IgM/IgG Combo Test Kit	^150^
	Human serum and acid citrate dextrose (ACD) plasma	Overall PPA: 97.14% NPA: 100%	Jiangsu Well Biotech Co., Ltd.	Lateral Flow	Orawell IgM/IgG Rapid Test	^151^
	Human serum, plasma, venous whole blood	PPA(0–7, 8–14, ≥15, Days from Symptom Onset): (1) IgG: 0%, 56.6%, 96.21% (2) IgM: 33.33%, 83.02%, 97.73% NPA: IgM:99.46%, IgG:100%	Biohit Healthcare (Hefei) Co. Ltd.	Lateral Flow	Biohit SARS‐CoV‐2 IgM/IgG Antibody Test Kit	^152^
Total Neutralizing Antibodies	Human serum and plasma	Total PPA: 96.0% (<7: NA, 8–14: 85.7%, >15: 97.7%, Days Between Onset of Symptoms and Specimen Draw) NPA: 98. 9%	ZEUS Scientific, Inc.	Indirect ELISA	ZEUS ELISA SARS‐CoV‐2 Total Antibody Test System	^153^
	Human serum and plasma	PPA: 96.2% (87.3–99.0%) NPA: 96.3% (89.8–98.8%)	InBios International, Inc.	Qualitative competitive inhibition ELISA	SCoV‐2 Detect Neutralizing Ab ELISA	^154^
	Human venous/fingerstick whole blood, plasma, serum	Serum and Plasma Samples: PPA (≤7, 8–14, ≥15, Days post‐RT‐PCR test): 94.9%, 96.0%, 100% NPA: 97.9%	NOWDiagnostics, Inc.	Lateral Flow (Colloidal Gold)	ADEXUSDx COVID‐19 test	^155^
	Human serum and plasma	LoB: 0.30 U/ml, LoD: 0.35 U/ml LoQ: 0.40 U/ml, PPA (0–7, 8–14, ≥ 15, Days after PCR positive result): 90.6%, 87.0%, 96.6%, NPA: 99.98%	Roche Diagnostics, Inc.	ECLIA	Elecsys anti‐SARS‐CoV‐2S	^156^
	Human serum and plasma	Overall PPA: 93.85% (0–7: 66.67%, 8–14: 92.31%, > 15: 95.92%, Number of days after symptom onset) NPA:97.83%	QIAGEN, GmbH	Digital lateral flow	QIAreach anti‐SARS‐CoV‐2 total test	^157^
	Human serum and plasma	Serum: PPA:98.86%; NPA:100% Plasma: PPA:100%; NPA: 95.6%	Bio‐Rad Laboratories, Inc.	ELISA	Platelia SARS‐CoV‐2 total Ab assay	^158^
IgG	Human serum	LoB: 0.029 μg/ml, LoD: 0.051 μg/ml, LoQ: 0.213 μg/ml, PPA (0–7, 8–14, ≥ 15, Days from positive PCR test): 45.16%, 87.50%, 100.00%, NPA: 99.19%	Quanterix Corporation	Paramagnetic microbead‐based sandwich ELISA	Simoa semi‐quantitative SARS‐CoV‐2 IgG antibody test	^159^
	Human serum, plasma	Total PPA: 92.7%(≤7: 75.8%, 8–14: 95.3%, ≥15: 96.8%, >18: 100%, Days between positive PCR and Sample Collection) NPA: 99.6%	Beckman Coulter, Inc.	CLIA	Access SARS‐CoV‐2 IgG	^160^
	Human serum	PPA(0–7, 8–14, ≥15, Days from positive PCR): 73.01%, 100%, 100% NPA (Two clinical studies): 97.68% (379/388), 94.4% (236/250)	Emory Medical Laboratories	ELISA	SARS‐CoV‐2 RBD IgG test	^161^
	Serum and plasma	PPA (0–7, 8–14, ≥15): (1) Days post‐symptom onset: 49.33%, 82.61%, 98.11% (2) Days post‐positive PCR: 56.07%, 95.77%, 97.56% NPA:99.55%	Abbott Laboratories Inc.	CMIA	AdviseDx SARS‐CoV‐2 IgG II	^162^
	Human serum and plasma	PPA(0–7, 8–14, ≥15, Days Post Agreement Symptom Onset): 61.9%, 92.9%, 100% NPA: 100%	Siemens Healthcare Diagnostics Inc.	CLIA	Dimension EXL SARS‐CoV‐2 IgG (CV2G)	^163^
	Human serum or plasma	PPA (≥15, Days post PCR confirmation): 93.3% NPA:99.2%	EUROIMMUN US, Inc.	ELISA	EUROIMMUN Anti‐SARS‐CoV‐2S1 Curve ELISA (IgG)	^164^
IgM	human serum and plasma	PPA (0–7, 8–14, ≥15, Days from Symptom Onset): 26.1%, 83.3%, 94.4% NPA:98.3%	Diazyme Laboratories, Inc.	CLIA	Diazyme DZ‐Lite SARS‐CoV‐2 IgM CLIA Kit	^165^

Abbreviations: LoB, limit of blank; LoD, limit of detection; LoQ, limit of quantitation; NPA, negative percent agreement, specificity; PPA, positive percent agreement, sensitivity.

### 4.1 Colloidal gold immunochromatography

The lateral flow assay (LFA)‐based colloidal gold immunolateral flow chromatography kit consists of an in‐line sample pad, a conjugate pad, an incubation and detection pad (test and control lines), and an absorption pad for serum, plasma and whole blood.^82^ The principle of operation is robust and simple, the sample (containing the test solution, buffer and functionalized colloidal gold particles, binding antibodies, antigens, and proteins) is added to the sample pad where it flows through the capillary to the absorbent pad, where colloidal gold particles bound to SARS‐CoV‐2 antigens can indirectly bind to IgG/IgM binding complexes and anti‐human IgG/IgM antibodies on the test line, and colloidal gold bound to antibodies (e.g., rabbit and mouse antibodies that can bind to colloidal gold) can also bind to the corresponding antibodies at the control line. Finally, three results indicating positive, negative, and invalid (false positive or false negative) can be obtained from the colors in the test and control lines.^83^, ^84^

After the outbreak, a rapid IgM antibody assay was designed and developed for SARS‐CoV‐2 virus detection, which requires only 10–20 μl of serum and can be completed within 15 min. A team of Chinese researchers developed a colloidal gold immunolateral flow chromatography device that can co‐detect IgG and IgM, achieving rapid detection in 15 min.^85^ Separate detections of IgG or IgM is not as effective as combined IgG/IgM detection, and in a study of 470 individuals using the S protein and N protein as antigens, IgG and IgM antibodies could be detected using a colloidal gold immunolateral flow chromatography device; the kit achieved a general sensitivity of 92.9% and a specificity of 98.7%.^86^ Antibody‐based serological assays also require paying attention to the timing of infection, which might have an impact on the results of the assay. Wang et al. used the SARS‐CoV‐2 IgM/IgG antibody kit (colloidal gold method) in infected and noninfected individuals and found sensitivities of 50%, 70%, 92.5%, and 97.5% at 1–3, 4–6, 7–9, and >9 days after admission. In addition, the titers of SARS‐CoV‐2 targeted IgG as well as IgM antibodies from positive samples increased with time of admission, with the positivity rate for both antibodies increasing from 50% to 92.5%.^87^

The viral load in SARS‐CoV‐2 patient specimens and changes in serum levels of specific antibodies can have important implications for serological assays; therefore, a number of investigators have evaluated and analyzed different serological assay kits. In a recent study, the performance and availability of seven different antigen detection kits were evaluated in unvaccinated patients recruited for the first time at six sites in Germany and Brazil, with Mologic (sensitivity: 90.1%, specificity: 100%), Bionote (sensitivity: 89.2%, specificity: 97.3%), Standard Q (sensitivity: 81.9%, specificity: 99%) meeting the WHO criteria for assay sensitivity and specificity (sensitivity >80%, specificity >97%). The results indicated high susceptibility in the first 3 days after symptom onset (≥87.1%) and in individuals with a viral load ≥6 log_10_ SARS‐CoV‐2 RNA copies/ml (≥88.7%).^88^ UK researchers recently evaluated 12 lateral flow immunoassay (LFA) kits that are used to detect antibodies of SARS‐CoV‐2. The sensitivity and specificity of the 12 LFAs were low 21 days prior to symptom onset; however, they all increased 21 days after the onset of symptoms, with specificities ranging from 74.3% to 99.1% for IgM/IgG, 82.9% to 100%, and IgM specificity ranged from 75.2% to 98%. The Bionote had the highest overall sensitivity (79.0%) and its sensitivity for IgM/IgG response reached 88.2% after >21 days of symptom onset.^89^ With the emergence of variant strains, Pickering et al. investigated the specificity and LoD of six rapid test kits, such as the Innova Rapid SARS‐CoV‐2 antigen test, and the Spring Healthcare SARS‐CoV‐2 antigen rapid test Cassette, the SureScreen‐V kit, the Encode kit, and the E25Bio rapid diagnostic test. The specificity, LoD, and sensitivity were measured for the assay kits, with both SureScreen‐V and Encode achieving 100% specificity and Innova achieving the highest overall sensitivity (89%) for clinical samples, rising to 95.5% and 98.6% when used on specimens with Ct values below 28 and Ct values below 25, respectively.^90^

### 4.2 Enzyme‐linked immunosorbent assay

ELISA is considered the gold standard for laboratory testing for SARS‐CoV‐2. Using serological samples, the S protein (consisting of the S1 and S2 subunits, and the RBD) and the N protein of the virus can be used as the major immunogens to assay for serum virus‐neutralizing antibodies in patients,^91^, ^92^ which can assay immunoglobulins of the virus in samples.^93^ ELISA for virus detection is based on the antigen–antibody complex structure and enzyme‐labeled antibodies, among which indirect ELISA and sandwich ELISA are the two most commonly used methods of detection.^94^The enzyme on the enzyme‐labeled antibody can catalyze the hydrolysis, oxidation, and reduction of the substrate to form a colored substance, which can be analyzed qualitatively by the naked eye or quantitatively by a spectrometer or other device,^95^ where the strength of the colored signal is proportional to the level of the antigen or antibody is detected.

The patient's antibody levels, as well as the SARS‐CoV‐2 protein as an antigen, are two important factors affecting serological testing. Most patients infected with the new coronavirus develop specific IgM, IgA, and IgG responses within days 5–15, with IgM and IgA lasting 3–6 weeks and IgG lasting several months.^96^, ^97^ Recently, an ELISA kit was developed using the RBD region from S protein, which had a specificity of 99.3% and could detect a large number of antibodies 2 weeks after the appearance of symptoms.^98^ ELISAs to assay IgG and IgM antibodies using the N and S proteins of the new coronavirus have been developed and the positive detection rates for the S protein‐based ELISA and the N protein‐based ELISA were 82.2% and 80.4%, with the S protein‐based ELISA being significantly more sensitive to IgM than the N protein‐based ELISA.^92^

### 4.3 Mutation sites on mutant strains cause antibody capture evasion in serological assays

The N protein is highly immunogenic and is the most produced protein by coronaviruses, and it can cause high titers of neutralizing antibodies in the humoral immune response and modulate the host cell immune response to accelerate the viral life cycle.^99^ Therefore, purified N proteins and their neutralizing antibodies are often used as markers to detect the corresponding antibodies or antigens in samples. In a study of 1441 subjects, researchers evaluated the Abbott PanbioTM COVID‐19 Ag rapid antigen detection kit with an overall specificity of 99.9% (95% CI: 99.5–100) and a sensitivity of 68.9% (95% CI: 55.6–79.8). The investigators found multiple disruptive amino acid substitutions in the 229–374 immunodominant epitopes of the viral N antigen by viral sequencing and sequence matching. These also included A376T coupled to M241I and the most common A220V mutation, which escaped detection by capture antibodies and gave false‐negative Abbott PanbioTM COVID‐19 Ag assay results.^100^

Given that the N antigen or “S antigen + N antigen” is mostly used as a marker in current serological kits, we point out that mutated sites in mutant strains may escape antibody capture, leading to reduced sensitivity and false‐negative results. In Omicron, for example, there are 32 mutant sites on the S protein, including N501Y, L452, K477, and E484, which have been shown to evade serum‐neutralizing antibody binding.^101^, ^102^, ^103^ For the “S antigen + N antigen” assay kit, the presence of a large number of mutations on the S protein can cause a significant decrease in assay sensitivity and lead to false‐negative results in serological assays. Therefore, we suggest that researchers evaluate and validate currently available antigen detection kits using VOCs samples and develop neutralizing antibodies based on conserved epitopes to improve the sensitivity of antigen detection kits.

### 4.4 Analysis of antigen–antibody‐based serological SARS‐CoV‐2 detection

In general, antigen–antibody‐based serological SARS‐CoV‐2 detections, such as Ag‐rapid detection tests (Ag‐RDTs) or antibodies specific tests, are more suitable for POC testing. And the ELISA, considered the gold standard for laboratory testing for SARS‐CoV‐2, always serves as a complementary technique for clinical diagnosis. Tali et al. analyzed five studies and summarized that the average sensitivity of Ag‐RDTs was found to be 56.2% (95% CI: 29.5%–79.8%) and the average specificity of 98.9% (95% CI: 97.3%–99.5%).^104^ Briefly, Ag‐RDTs possess high specificity like molecular diagnostic methods. In contrast, low sensitivity is a disadvantage that cannot be neglected. This defect is associated with the type of specimen,^105^ time of specimen collection,^106^ antigens stability,^107^ and quality of the specimen. Mertens et al. reported that viral loads of specimens made a great difference to Ag‐RDTs sensitivity. When viral loads were high (real‐time RT‐PCR CT values of <25), the sensitivity of Ag‐RDTs achieved 74.8%. However, the overall sensitivity was only 57.6% when all specimens were taken into consideration.^108^ Therefore, WHO suggests that Ag‐RDTs tend to conditions that are remote and underserved or seriously pandemic. Given the average time of immune response to SARS‐CoV‐2 is around 1–2 weeks, the span of immune response will influence the clinical diagnosis. In the post‐pandemic era, vaccination will gradually cover most people, which will complicate the results of antibody detection. Therefore, the applicable conditions of antibody detection should be considered (Table 3).

## 5 MULTI‐CHANNEL DETECTION OF SARS‐COV‐2 AND OTHER RESPIRATORY INFECTIOUS DISEASES

In the context of the SARS‐CoV‐2 pandemic, other respiratory infectious diseases cannot be ignored, such as influenza A/B and respiratory syncytial virus (RSV). The clinical signs and symptoms of these respiratory infectious diseases are similar to that of SARS‐CoV‐2. Therefore, developing multi‐channel detection assays is significant. Wang et al developed an ultrasensitive fluorescent immunochromatographic assay based on multilayer quantum dot nanobead for simultaneous detection of SARS‐CoV‐2 antigen and influenza A virus,^109^ which showed excellent sensitivity and specificity compared to traditional AuNP‐based ICA method and ELISA kits. Apart from serological multi‐channel test assays, the majority of test methods rely on nucleic acid detection. Zhou et al developed simultaneous detection of SARS‐CoV‐2, influenza, and Respiratory Syncytial Viruses (RSV) based on the CRISPR‐Cas12 system.^110^ They demonstrated that CRISPR‐Cas12a with specific gRNAs had an LOD of 1 copy/μl for SARS‐CoV‐2 and 100 copies/μl for influenza A and B and RSV, respectively. The CRISPR‐Cas12a test produced 100.0%, 93.8%, 100.0%, and 90.0% sensitivity for SARS‐CoV‐2, influenza A, influenza B, and RSV, respectively, with a specificity of 100%. All these tests required 30 min at one time. Recently, the Allplex™ SARS‐CoV 2/FluA/FluB/RSV (SC2FabR) assay was reported for the simultaneous detection of four viruses. Via comparison of four commercially available kits (the Allplex™ 2019‐nCoV kit, Standard M n‐CoV Real‐Time Detection kit, Allplex™ Respiratory panel 1 kit, and Advansure™ RV‐plus Real‐Time RT‐PCR kit), the sensitivity of SC2FabR was 100% (99/99) for Flu A, 100% (91/91) for Flu B, and 98.7% (74/75) for RSV, with 100% specificity for all targets compared with that of the RP1 assay. Besides, the sensitivity of the SC2FabR assay was 99.0% (98/99) for Flu A, 100% (91/91) for Flu B, and 92.0% (69/75) for RSV, and the specificity levels were 99.5% for Flu A and RSV and 99.7% for Flu B compared with RV‐plus assay.^111^

## 6 ULTRA‐SENSITIVE QUANTUM DOTS AND BIOSENSORS: A NEW HIGH‐PERFORMANCE POCT KIT THAT COMBINES QUALITATIVE AND QUANTITATIVE DETECTION

In the face of the rapidly spreading SARS‐CoV‐2 epidemic and the emerging class of highly infectious mutants that evade antibody capture traditional serological tests are not ideal. ELISA, although a widely used laboratory serological test, requires a long assay process of 2 h due to the incubation and washing operations involved.^91^ The results of colloidal gold‐based lateral flow kits are unreliable due to their different evaluation criteria and quality are again unreliable, and the test is qualitative and does not allow quantitative analysis of the extent of antibody response in patients.^112^ Therefore, we present here a kit for the use of novel ultrasensitive quantum dots and biosensors in SARS‐CoV‐2 assay and the related performance to achieve a complementary quantitative antigen/antibody assay.

### 6.1 Application of ultrasensitive quantum dots for rapid quantitative detection in POCT

Quantum dots are novel engineered nanomaterials with outstanding optoelectronic properties that are applied to ultrasensitive detection in bioanalysis, diagnostics, and imaging strategies. In recent years, the functionalization of QDs with different biomarkers, such as antigens, antibodies, nucleic acids, and peptides, show great potential for clinical diagnosis.^113^

Quantum dots can couple antibodies by specific and nonspecific labeling.^114^ The specific labeling uses the directional coupling agent Maleamide–polyethylene glycol–succinimide ester (SMPEG) to couple the quantum dots and antibodies. And the nonspecific labeling uses EDC/NHS chemistry methods to conjugate the QDs and antibodies.^115^

Wang et al. developed a new ICA method by using a novel silica‐QD nanocomposite with triple‐QD shell (SiTQD) as the advanced signal probe. This SiTQD nanocomposite with a triple QD‐shell is constructed by PEI‐mediated LBL self‐assembly. Then making the SARS‐CoV‐2 NP antigen detecting antibody conjugated with SiTOD NPs via carbodiimide chemistry. Compared with previous ICA methods, three layers of quantum dots greatly enhanced the fluorescence signal. And high‐performance SiTOD ensures this system with high stability and sensitivity.^109^Wang et al. first developed two‐channel ICA to simultaneously detect SARS‐CoV‐2 and FluA. Under the optimal conditions, the LOD values for SARS‐CoV‐2 NP and H1N1 were estimated as 5 pg/ml and 50 pfu/ml by quantitative analysis of throat swab samples. However, the LODs determined by the ELISA kits for SARS‐CoV‐2 NP and H1N1 was 0.1 ng/ml and 5000 pfu/ml, respectively. Thus, they demonstrated that the sensitivity of SiTOD‐ICA was 100 times higher than the traditional AuNP‐based ICA method and over 20 times that of ELISA kits. Besides, compared with two quantum dot assays (SiQD and SiDQD‐based ICA), the fluorescence images of SiTQD‐based ICA were twice than two other quantum dots assays in different concentrations (10–0.1 ng/ml). Therefore, the fluorescent immunochromatographic assay based on multilayer quantum dot nanobead can be an efficient POCT tool for rapidly and accurately detecting SARS‐CoV‐2 or other pathogens. Zhang et al. combined the CRISPR‐Cas13 system with fluorescent quantum dot nanobead SARS‐CoV‐2 (CFNS) assay.^116^ The CRISPR/Cas13 reaction could specifically be recognized and cleaved the amplified products. Then the cleavage products and sheep anti‐FITC IgG antibody‐labeled quantum dot microsphere (QDM‐anti‐FITC antibody) would be mixed and added to the test strip. The fluorescence detector could show the fluorescence ratio to get the results. Compared to different Ct values of RT‐PCR with this method, they found that the results detected by the CFNS assay have a linear relationship with the results of the golden standard, which means CFNS could get reliable results in less time. Via detection of standard positive RNA at different concentrations from 1015 copies/ml to 1 copy/ml, they demonstrated that CFNS could reach the detection limit of 1 copy/ml In general, the novel test methods based on quantum dot nanobead are fast, sensitive, specific and easy to operate, which is more suitable for POCT compared with ELISA or traditional immunolateral flow chromatography methods.

Absolutely, with the global epidemic of the SARS‐CoV‐2, the treatment diagnosis and monitoring of patients is more critical. However, current testing methods often require a great number of professional laboratory operators and manual entry of test results, which adds a lot of pressure to the already heavy burden of the healthcare system. Therefore, it is significant to develop visualization devices and construct data integration platforms. Zhang et al. incorporated smartphones and quantum dot microbead assay to monitor pandemics in real‐time.^117^ On the one hand, quantum dot microbead improves the sensitivity and specificity of virus detection, on the other hand, handheld detector enables device portability and data sharing.

### 6.2 Biosensor for SARS‐CoV‐2 antigen and antibody detection

Currently, biosensors are mainly based on field‐effect transistors (FETs) and surface plasmon resonance (SPR) principles, and both FETs with the aid of graphene coating and SPRs relying on electron resonance on precious metal surfaces can be used to detect protein–protein, antigen–antibody, and protein–nucleic acid interactions,^118^, ^119^ and to track biomarkers such as antigens, antibodies, nucleic acids, and ROS.^120^ Elledge et al. developed the COVID‐19 FET sensor, which was sprayed with antibodies specific for S protein on graphene, to capture SARS‐CoV‐2 antigen in nasopharyngeal swab specimens and measured a LOD of 2.42 × 10^2^ copies/ml in validated clinical samples. A protein engineering‐based approach has been developed to design a simple luciferase (spLUC) antibody sensor that can analyze serum, plasma, whole blood, and saliva samples within 30 min to generate quantitative serological data. spLUC sensor sensitivity for detecting antibodies to S protein was shown to be 89% by testing over 150 patient samples and 98% sensitivity for detecting antibodies to N protein, with specificity exceeding 99% for both. Notably, Elledge et al. used a modular design approach in the development process that allows for flexibility in responding to mutant RBD structural domains of emerging VOCs and evaluating antibody responses to emerging variants.^121^ Three SARS‐CoV‐2 specific single chains were screened by phage display technology constructs mutable fragment crystallizable fragment (scFv‐Fc) fusion antibodies, the developers developed a cellulose nanobead (CNB)‐based LFIA biosensor that can specifically detect SARS‐CoV‐2 N protein in 20 min with a detection line of 2 ng of antigenic protein, and the results can be analyzed qualitatively by color‐displayed bands or by a handheld portable LFIA reader in quantitative analysis results can be obtained within 10 s, enabling home telemedicine monitoring.^122^

## 7 CLOUD PLATFORM FOR EPIDEMIC PREVENTION AND CONTROL: TELEMEDICINE TESTING KITS AND MOBILE DEVICES IN EPIDEMIC TRACKING AND CONTROL

In the post‐COVID‐19 era, it is increasingly important to facilitate patients to obtain faster and more convenient medical services and dynamically monitor the spread of the epidemic. In other words, how to integrate medical data and predict the development of epidemic situation in the future maybe make a real difference to control the epidemic. The Internet of Medical Things (IoMT) promote the proactive tele‐healthcare of suspected COVID‐19 patients.^123^ With the development of 5G technology, Guo et al reported a 5G‐enabled fluorescence sensor for rapid detection and tele‐monitoring of COVID‐19 patients.^124^ Not only can the fluorescence sensor detect the strip in 10 min, but also connect to edge hardware devices (personal computers, smartphones, IPTV, etc.) and the fog layer of the network to perform reliable data transmission with low latency and high security. What's more, several COVID‐19 monitoring mHealth applications were proposed, which enabled patients to record and upload their results.^125^ In the online hyper‐connected world, the SARS‐CoV‐2 epidemic can be predicted through the sharing and analysis of medical data, including mathematical prediction models and algorithms (Figure 3b).

In conclusion, with the use of fast and accurate POC biosensing equipment, the detection results are uploaded to the mobile cloud monitoring platform in real time, which in turn establishes a cloud‐based big data quality management and epidemic spread control system, generating a dynamic map of virus epidemic development control from two dimensions, spatial and temporal, so that the CDC command center can fully and timely understand the instantaneous information changes of the epidemic prevention and control grassroots units to achieve efficient and rapid linkage and unified Coordinated scheduling and resource allocation, thus effectively controlling the epidemic.

## 8 CONCLUSION AND PROSPECT

At present, a range of nucleic acid molecule and antigen–antibody based methods are accessible for SARS‐CoV‐2 detection. The highly specific and sensitive nature of nucleic acid testing has led to its use in many countries for high‐throughput analysis of numerous specimens in the population; but because of its equipment, space, and personnel requirements, nucleic acid testing can only be performed in specialized sites such as hospitals and CDCs. Serology‐based test kits can meet the need for home and community‐based POC testing because of their small size, flexibility, and less demanding testing environment. The recently emerged antigen–antibody test kits with high sensitivity and specificity can also serve as a supplement to detect SARS‐CoV‐2 outbreaks caused by strong mutant strains, such as Delta and Omicron, as well as for outbreak control in home and community care settings.

qRT‐PCR continues to be the mainstream gold standard method to detect SARS‐CoV‐2 qualitatively and quantitatively. Nevertheless, the assay still has limitations, such as differences in viral load in various samples that affect the sensitivity of the assay, and mutation sites generated in mutant strains that affect the binding of primers and detection antibodies in serological kits. Highly infectious SARS‐CoV‐2 mutants and asymptomatic patients with false‐negative test results also present a requirement for fast, highly sensitive, highly specific, and cost‐effective POC‐based testing kits. LAMP‐ and CRISP/Cas‐based POC assays have been rapidly developed, with the results of both kits being available in 0.5–1 h. Moreover, the design of relevant primers and guide RNAs allows for flexible detection of mutant strains as the mutant genome is sequenced and common SARS‐CoV‐2 mutation loci are analyzed. LAMP is compatible with many different types of LFA (e.g., colloidal gold immunochromatography kits) that have been widely used in the United States and Europe. However, POC‐based detection kits have not yet achieved widespread popularity in some poor and developing countries and regions (e.g., Africa). In China, although RT‐PCR is mainly used to detect infection, the China National Health Commission recently issued documents related to the new coronavirus self‐test system, advocating people to adopt self‐testing to ease the pressure of controlling the epidemic.

The SARS‐CoV‐2 virus mutant strain pandemic represented by Delta and Omicron has proved the value of rapid detection kits. In the future, POCT test kits with easy operation, fast detection speed, and high specificity and sensitivity will become the mainstream of analysis and are expected to effectively screen infected individuals at home and in the community to control mutant strain outbreaks. Unlike time‐consuming and expensive whole‐genome sequencing to identify SARS‐CoV‐2 variants, flexible gRNA and primer design for high‐performance CRISP/Cas and RT‐LAMP kits are expected to diagnose and track strongly infectious mutants such as Delta and Omicron in a timely manner in the future, so that epidemic prevention policies and treatment plans can be formulated according to the hierarchy of different infectious mutant strains and Rational allocation of medical resources. Although serology‐based rapid antibody tests can enable large‐scale immune screening, they still have a lag and cannot prove the presence of the virus.

Antigen detection is expected to move the detection window forward for early screening. Currently, N and S proteins are often used as markers in the assay, but due to the generation of mutant strains and their mutation sites, antibody capture escape often occurs in the assay, especially with kits that use S proteins, the sensitivity of the assay will be significantly reduced. In the future, the development of recombinant antibodies based on conserved sites, the use of ultrasensitive quantum dot materials, and the application of modularly designed biosensors are expected to circumvent the risk of escape.

Therefore, we suggest that until the emergence of vaccines with efficient cross‐protection and clinically validated therapeutic regimens, developers need to focus on rapid antigen detection devices and, with a large number of clinical samples to validate them, develop high‐performance POCT kits that can be used at the point of care, such as colloidal gold, ultrasensitive quantum dots, and biosensors, and use new nanoparticles and other materials to effectively move the detection window of infection forward, expand the scope of application of the kit by combining it with readable home devices such as smartphones, and realize timely tracking of strongly infectious mutant strains such as Delta and Omicron using flexibly designed POCT kits such as CRISP/Cas and RT‐LAMP to control the epidemic in households and communities in a timely manner so that appropriate actions can be taken to effectively control the SARS‐CoV‐2 and its mutant strains in the future.

## AUTHOR CONTRIBUTIONS

**Yuxuan Zhang:** Writing – original draft (lead); writing – review and editing (lead). **Zhiwei Huang:** Writing – original draft (equal). **Jiajie Zhu:** Visualization (equal). **Chaonan Li:** Formal analysis (equal). **Zhongbiao Fang:** Software (supporting). **Keda Chen:** Conceptualization (equal); data curation (equal); writing – review and editing (supporting). **Yanjun Zhang:** Conceptualization (equal); data curation (equal).

## CONFLICT OF INTEREST

The authors have no conflicts of interest to declare.

### PEER REVIEW

The peer review history for this article is available at https://publons.com/publon/10.1002/btm2.10356.

## Data Availability

Some or all data, models, or code generated or used during the study are available from the corresponding author by request.
